# Exposure-response analyses of venetoclax combined with hypomethylating agents in myelodysplastic syndromes: a retrospective study

**DOI:** 10.3389/fphar.2025.1586910

**Published:** 2025-07-14

**Authors:** Ji-Xin Tian, Ping Zhang, Xiao-Xu Wang, Jin-Wen Li, Dong-Xue Liu, Yi-Ying Liu, Zhi-Jian Xiao, Wen-Juan Miao

**Affiliations:** State Key Laboratory of Experimental Hematology, National Clinical Research Center for Blood Diseases, Haihe Laboratory of Cell Ecosystem, Institute of Hematology and Blood Diseases Hospital, Chinese Academy of Medical Sciences and Peking Union Medical College, Tianjin Institutes of Health Science, Tianjin, China

**Keywords:** venetoclax, exposure-response, hypomethylating agents, myelodysplastic syndromes, therapeutic drug monitoring

## Abstract

**Background:**

Venetoclax (VEN), an orally bioavailable B-cell lymphoma-2 inhibitor, shows promising activity in patients with myelodysplastic syndromes (MDS) when combined with hypomethylating agents (HMAs). However, research regarding the VEN exposure in Chinese patients with MDS remains notably sparse.

**Methods:**

This study retrospectively collected the predose (C_0_) and 6 h post-oral dosing plasma concentration (C_6_) of VEN for exposure-response analyses, using graphical analysis, receiver operator characteristic (ROC) curves, and logistic regression. Sixty-four patients were included in the exposure-safety analyses. Thirty-nine patients who were treated with HMAs as first-line treatment and added VEN within 4 cycles, or received VEN + HMAs as the initial treatment, were included in the exposure-efficacy analyses.

**Results:**

In Chinese MDS patients, the average C_0_ and C_6_ of VEN were 1990.60 ± 1,591.12 ng/mL and 2,966.66 ± 1,406.96 ng/mL, respectively, with large interindividual variability. The use of azole antifungals was a significant factor influencing VEN concentration (*P* < 0.05). Compared to VEN 400 mg administered without azole antifungals, concomitant use of azole antifungals with VEN 100 mg resulted in a 100.03% and 18.50% increase in VEN C_0_ and C_6_, respectively. In the efficacy analyses, the combination of VEN and HMAs achieved an overall response rate of 69.23%. Based on logistic regression and ROC curve analyses, the peak plasma concentration of VEN, without dose normalization, exhibits a significant correlation with treatment success (*P* < 0.05). Other factors, including C_0_, demographics, and disease characteristics (e.g., molecular mutations, baseline grade III/IV neutropenia, and prior therapies), were not associated with the probability of marrow remission. In the safety analyses, higher VEN concentrations were not associated with an increased probability of grade ≥3 infection or a more serious decrease in platelets and neutrophils.

**Conclusion:**

This study offers a preliminary exploration of the potential exposure-efficacy and exposure-safety relationships of VEN combined with HMAs for the treatment of MDS in Chinese patients. Given the interindividual variability in VEN exposure, therapeutic drug monitoring is recommended as an essential part of clinical practice in MDS treatment. Future research is necessary to conduct more in-depth and large-scale analyses to determine the optimal exposure threshold for VEN.

## 1 Introduction

Myelodysplastic syndromes (MDS) are a group of clonal hematological disorders with a high risk of conversion to acute myeloid leukemia (AML). Allogeneic hematopoietic stem cell transplantation (allo-HSCT) is the only known curative treatment of MDS. Unfortunately, most patients cannot undergo allo-HSCT because of advanced age and/or comorbidities. The primary therapy for the MDS patients who are ineligible for allo-HSCT is hypomethylating agents (HMAs), but the overall results are poor, with a 5-year survival rate of approximately 31% (Zeidan et al., 2019).

In recent years, a greater understanding of the molecular mechanisms underlying MDS has been achieved. *In vitro* data suggest that MDS acquires resistance to apoptosis by leveraging the B-cell lymphoma-2 (BCL-2) family proteins (Jilg et al., 2016). BCL-2 family is a key apoptotic factor in the endogenous apoptotic signaling pathway that inhibits apoptosis and plays an important role in tumorigenesis and development (Pan et al., 2014). Venetoclax (VEN) is a potent, selective oral inhibitor of BCL‐2 that blocks the activity of the pro‐survival BCL‐2 protein, primes neoplastic cells for apoptosis, and exhibits considerable synergy with HMAs (Tsao et al., 2012; Ball et al., 2020; Bazinet et al., 2022; Zeidan et al., 2023; Wei et al., 2019).

VEN was approved for marketing in China at the end of 2020. Due to the short period since its approval, there is little experience in clinical application, especially in MDS treatment. VEN is primarily metabolized by cytochrome P450 3A (CYP3A), and its exposure is largely influenced by food intake or moderate-to-strong CYP3A inhibitors (e.g., triazoles), presenting a high risk of drug-drug or food-drug interactions (Salem et al., 2017). Studies have shown that the pharmacokinetic parameters of VEN exhibit a racial difference: in Chinese subjects, the peak plasma concentration of VEN (5–8 h after oral administration (Deeks, 2016)) was 94% higher than in non-Asian subjects receiving the same dose (Cheung et al., 2018). In clinical practice, real-world data have highlighted a great pharmacokinetic interindividual variability, and it remains uncertain whether this variability affects efficacy (Yang et al., 2022; Kobayashi et al., 2022; De Gregori et al., 2023; Wang et al., 2024; Philippe et al., 2024). On the other hand, the most commonly reported toxicity of VEN is myelosuppression, which often leads to dose reduction or treatment discontinuation. (Wei et al., 2019). MDS is defined by bone marrow dysplasia and cytopenia, and the addition of VEN to the HMAs regimen may lead to cumulative myelosuppression (Ball et al., 2020). However, the relationship between VEN exposure and efficacy/safety remains controversial (Agarwal et al., 2019; Brackman et al., 2022; Samineni et al., 2022; Parikh et al., 2018; Freise et al., 2017). As such, the aims of the current study were to 1) analyze the plasma concentrations of VEN in Chinese MDS patients and their influencing factors, with a particular focus on the impact of azole antifungals, and 2) explore the exposure-efficacy/safety relationships of VEN + HMAs in Chinese MDS patients. These endeavors will facilitate the development of personalized VEN + HMAs therapies.

## 2 Methods

### 2.1 Study population

The study population comprised MDS patients between October 2021 and November 2023 at the Institute of Hematology and Blood Diseases Hospital. The inclusion criteria were as follows: (1) diagnosis of MDS based on criteria (Arber et al., 2016; Greenberg et al., 2012; Pfeilstöcker et al., 2016); (2) treatment with at least one cycle of VEN plus HMAs; (3) at least one bone marrow follow-up, and (4) at least one determination of VEN concentration.

Information including demographic data, disease characteristics, laboratory results, details of prior therapies, cytogenetic and molecular alterations at the time of MDS diagnosis was collected. MDS types and risk stratification were classified according to the World Health Organization 2016 criteria and 2012 Revised International Prognostic Scoring System (IPSS-R), respectively (Arber et al., 2016; Greenberg et al., 2012).

Patients who were treated with HMAs as first-line treatment and added VEN within 4 cycles or received VEN + HMAs as the initial treatment were included in the efficacy assessment. All the patients who met the inclusion criteria were included in the safety assessment. We obtained information on patients’ responses and adverse events (AEs) to VEN + HMAs combination therapy.

This was a retrospective study in which TDM of VEN was part of routine clinical practice. Dosage modifications of VEN were not based on the concentrations, which were used only as a reference for treatment decisions.

The study was conducted in accordance with the Declaration of Helsinki (as revised in 2013) and approved by the Ethics Committee of Blood Diseases Hospital, Chinese Academy of Medical Sciences (No. IIT2021029-EC-1). The off-label use of VEN was conducted under the oversight of the Pharmacy and Therapeutics Committee of Blood Diseases Hospital, Chinese Academy of Medical Sciences. All procedures were in compliance with ethical standards. Patients were fully informed about the off-label use of VEN, including its potential benefits and risks. Informed consent was obtained from all patients or their legal representatives prior to the administration of the drug. The requirement for individual consent for this retrospective analysis was waived.

### 2.2 Treatment

VEN-based regimens were administered as follows: VEN (VENCLEXTA^®^, AbbVie Inc., 100 mg) was taken within half an hour after meals at a dose of 400 mg/d for 14 days and was reduced to 7–12 days if the patient had excessive treatment-related myelosuppression, and the dosage was adjusted accordingly at the discretion of the treating physicians. Patients receiving strong or moderate CYP3A inhibitors, such as azole antifungals, received 100 mg VEN.

Azacitidine (Xinsen^®^, Jiangsu Hansoh Pharmaceutical Group Co. Ltd., 100 mg) was administered subcutaneously at a dose of 50–75 mg/m^2^/d on days 1–7 of each 28-day cycle, and decitabine (Aodixi^®^, Jiangsu Aosaikang Pharmaceutical Co. Ltd., 50 mg) was administered intravenously at a dose of 20 mg/m^2^/d on days 1–5 of each 28-day cycle. Cycles beyond the first could be variably delayed to allow peripheral count recovery.

Routine blood tests, liver and kidney function tests, electrocardiograms, and myocardial enzymes were regularly monitored. Blood transfusion was administered as clinically indicated. Patients showing a clinical response continued to receive VEN + HMAs until disease progression, life-threatening adverse reactions, or allogeneic hematopoietic stem cell transplantation (allo-HSCT).

### 2.3 Efficacy and safety assessments

According to the 2006 modified International Working Group (IWG) response criteria for MDS, complete remission (CR), partial response (PR), marrow CR (mCR), stable disease (SD), and progressive disease (PD) were used to assess treatment response (Cheson et al., 2006).

AE data were graded according to the National Cancer Institute Common Terminology Criteria for Adverse Event (CTCAE) version 5.0 (National Cancer Institute, 2017). Hematological AEs were defined as follows: platelet counts between 25 and 50 × 10^9^/L were indicative of grade 3 thrombocytopenia, while counts below 25 × 10^9^/L were indicative of grade 4 thrombocytopenia. Neutrophil counts between 0.5 and 1.0 × 10^9^/L were indicative of grade 3 neutropenia, while counts below 0.5 × 10^9^/L were indicative of grade 4 neutropenia. Patients were considered to have grade 3 or higher infections if they required intravenous antibiotic, antifungal, or antiviral therapy.

### 2.4 VEN plasma concentration measurement

Plasma concentrations of VEN were measured using an ultra-high performance liquid chromatography tandem mass spectrometry (UPLC-MS/MS). The UPLC-MS/MS system was comprised of an ACQUITY UPLC I-Class separation module (Waters, USA) coupled to a Xevo TQD micro triple-quadrupole mass spectrometer (Waters, USA) in positive electrospray ionization (ESI^+^) mode, and chromatographic separation was conducted on an ACQUITY UPLC BEH C_18_ column (2.1 × 50 mm, 1.7 µm, Waters), maintained at 45°C with a gradient elution (0–1.5 min, 50%–75% of B; 1.5–2.0 min, 75%–100% of B; 2.0–2.5 min, 100%–50% of B, 2.5–4.0 min, 50% of B) at 0.5 mL/min, where mobile phases A and B were 0.1% formic acid +2 mM ammonium acetate in water and 0.1% formic acid in methanol, respectively. The autosampler temperature was set at 15°C. Under these conditions, VEN and [^2^H_8_]-VEN (internal standard, IS) were typically eluted at 1.80 and 1.74 min. Analytes were quantified in multiple-reaction monitoring (MRM) mode at *m/z* transitions of 868.6→321.3 for VEN and 876.6→329.3 for IS, respectively. The source temperature was set at 150°C and the capillary voltage at 3.5 kV, nitrogen was used as desolvation gas (800 L/Hr) at 350°C, and the fragmentor voltage and collision energy were optimized and set at 50 V and 42 V. Data acquisition and processing were controlled by Waters MassLynx software (version 4.1).

Blood samples for VEN were collected at steady state prior (C_0_) and 6 h after (C_6_) dosing (within a ±10 min window) and stored in EDTA-K_2_ anticoagulant tubes. The tubes were centrifuged at 3,500 rpm for 5 min to separate plasma. Then, 100 µL of plasma was aliquoted into an Eppendorf tube, and the IS solution was added. Subsequently, the samples were processed by protein precipitation, followed by vortexing (5 min) and centrifugation (14,000 rpm, 5 min). In total, 200 µL of the supernatant was transferred to UPLC vials and 10 µL was injected into the UPLC column.

The validation of the analytical method was based on the United States Food and Drug Administration (FDA) guidelines including calibration curve, precision, accuracy, selectivity, matrix effect, recovery, carry-over, and stability (U.S. Department of Health and Human Services, 2018).

### 2.5 Statistical analysis

All analyses were performed using the SPSS software (SPSS, Chicago, IL, United States). Each continuous variable was assessed for normality using the Shapiro-Wilk test. Normally distributed variables were compared using Student’s *t*-test. The changes between the levels before (C_0_) and after VEN administration (C_6_) were evaluated using the paired *t*-test. Non-normally distributed variables were compared using the Mann-Whitney *U* test. Pearson’s test was used to correlate normally distributed data; otherwise, Spearman’s correlation analysis was performed. Linear regression analysis was used to identify the factors contributing to the variability in VEN concentration. These tests were two-tailed, and a *P*-value below 0.05 was assumed to represent statistical significance. Patient characteristics were summarized with descriptive statistics using percentages for categorical variables.

### 2.6 Exposure‐response analyses

#### 2.6.1 Graphical analyses

The relationships between VEN exposure (C_0_ and C_6_) and both efficacy responses and safety events were first explored graphically using quartile and binary plots. The primary objective was to identify whether there were exposure-response relationships with the observed data. For the exposure-efficacy analyses, the response variable was the probability of CR + mCR + PR. For the exposure-safety analyses, the response variables were the probability of grade 3 or worse infection and the maximum change from baseline of neutrophils and platelets.

Comparison of the proportions of patients with efficacy and infection outcome across VEN concentration quartiles or binaries was performed using the Fisher exact test, and the Kruskal-Wallis test was used to compare the safety continuous variables across quartile groups.

#### 2.6.2 Receiver operator characteristic (ROC) curves

ROC curve analysis was used to identify VEN concentration thresholds associated with efficacy and safety outcomes during therapy. The efficacy and safety endpoints were evaluated as binary data. The area under the ROC curve (AUC) was calculated as an overall performance indicator of the blood level. Optimal thresholds were chosen using the Youden index, which maximizes the sum of the specificity and sensitivity of the ROC curve. (Youden, 1950).

#### 2.6.3 Logistic regression analyses

Logistic regression analyses were conducted to identify the covariates that may influence the efficacy of VEN in MDS patients. The efficacy endpoint was evaluated as the binary data (CR/mCR/PR, SD/PD). The covariates were identified based on scientific interest or prior knowledge of any possible relationship with efficacy, including body mass index (<25/≥25), age (<60/≥60), gender (male/female), grade III/IV neutropenia at baseline (yes/no), presence of TP53, DNMT3A, RUNX1, ASXL1, DDX41 mutation (yes/no), presence of three or more molecular mutations (yes/no), VEN exposure parameters (<ROC cut-off point/≥ROC cut-off point), dose-normalized C_6_ (C_6_/dose), the dosage of VEN, coadministration of azole antifungals, and prior therapies (yes/no). Covariate selection was performed via univariate analysis using a significance level of 0.2 as a requirement for continued inclusion in the multivariable logistic regression analysis. A joint examination of the explanatory factors by multivariate analysis was made, checking for collinearity. Last, the stability of the final model was assessed by bootstrap resampling (n = 1,000 replicates).

## 3 Results

### 3.1 Patients’ characteristics

This study included 64 patients; their demographic and clinical characteristics are shown in Table 1. The median age of the patients was 59 years (range, 23–74), and 37/64 (57.81%) were male. MDS with excess blasts-2 (MDS-EB-2) was the most frequent diagnostic classification (44/64, 68.75%), followed by MDS-EB-1 in 15 patients (23.44%). The most frequent mutations were TP53 (16/64, 25%) and ASXL1 (14/64, 21.88%), followed by DNMT3A (11/64, 17.19%), RUNX1 (10/64, 15.62%), DDX41 (10/64, 15.62%), and U2AF1 (9/64, 14.06%) (see Supplementary Figure S1). Regarding risk stratification characteristics, although the definition of higher-risk MDS is variable across clinical trials and routine clinical practice settings, an IPSS-R score of >3.5 is frequently used as a threshold to distinguish lower-risk MDS and higher-risk MDS (Greenberg et al., 2012). In this study, all included patients were categorized as higher-risk MDS.

**TABLE 1:** Patient characteristics (*N* = 64).

Characteristic	N	%
*Gender*		
Male	37	57.81
Female	27	42.19
*Age*		
<60 years	34	53.12
≥60 years	30	46.88
*Body Mass Index (BMI)*		
BMI < 25	30	46.88
BMI ≥25	34	53.12
*Prior therapies*		
0	42	65.63
≤3	7	10.94
>3	15	23.44
*Cycle of VEN + HMAs (When concentrations collected)*		
≤3	59	92.19
>3	5	7.81
*MDS type*		
EB-1	15	23.44
EB-2	44	68.75
Others	5	7.81
*IPSS-R*		
Intermediate (>3.5, ≤4.5)	8	12.50
high risk (>4.5, ≤6)	26	40.62
very-high risk (>6)	30	46.88
*Molecular mutation (Only high-frequency mutations are included)*		
DNMT3A	11	17.19
RUNX1	10	15.62
TP53	16	25
ASXL1	14	21.88
DDX41	10	15.62
U2AF1	9	14.06
NPM1	6	9.38
SRSF2	5	7.81

Abbreviations: VEN, venetoclax; HMAs, hypomethylating agents; IPSS-R, revised international prognostic scoring system; EB, excess blasts.

Among the 64 patients, 42 patients used VEN + HMAs as the initial treatment plan when they were first diagnosed with MDS, 7 patients had already received 1–3 cycles of HMAs protocol at the time of VEN initiation, and 15 patients experienced more than 3 cycles of prior regimens, including drugs undergoing clinical trials, such as the anti-T cell immunoglobulin and mucin-domain-containing-3 monoclonal antibody, selective inhibitor of NEDD8-activating enzyme (pevonedistat), and selective inhibitor of nuclear export (eltanexor).

In this study, 81.25% of patients received azacitidine and 18.75% of patients received decitabine in combination with VEN. Among the 52 patients who underwent efficacy assessment, the overall response rate (ORR) was 67.31% (35/52), including 33 patients (63.46%) who achieved CR or mCR and 2 patients (3.85%) who achieved PR. At the median follow-up time of 3.5 (range, 0.37–27.53) months, 61 patients (95.31%) had discontinued treatment. Reasons for treatment discontinuation were disease progression (n = 15; 23.44%), no response to treatment (n = 4; 6.25%), unacceptable toxicity (due to prolonged pancytopenia or severe pneumonia, n = 5; 7.81%), HSCT (n = 12; 18.75%) or loss to follow-up (n = 24; 37.50%). The median number of cycles of VEN + HMAs treatment was 2 (range 1–15), and the median number of cycles to disease progression for VEN + HMAs treatment was 5 (range 1–15).

### 3.2 VEN concentrations and influencing factors

The developed UPLC–MS/MS method was applied to measure VEN concentrations in plasma. The concentrations of VEN in 59 patients were collected from 1–3 cycles after the start of VEN + HMAs treatment, with only 5 cases having a collection time greater than 3 cycles.

**FIGURE 1:**
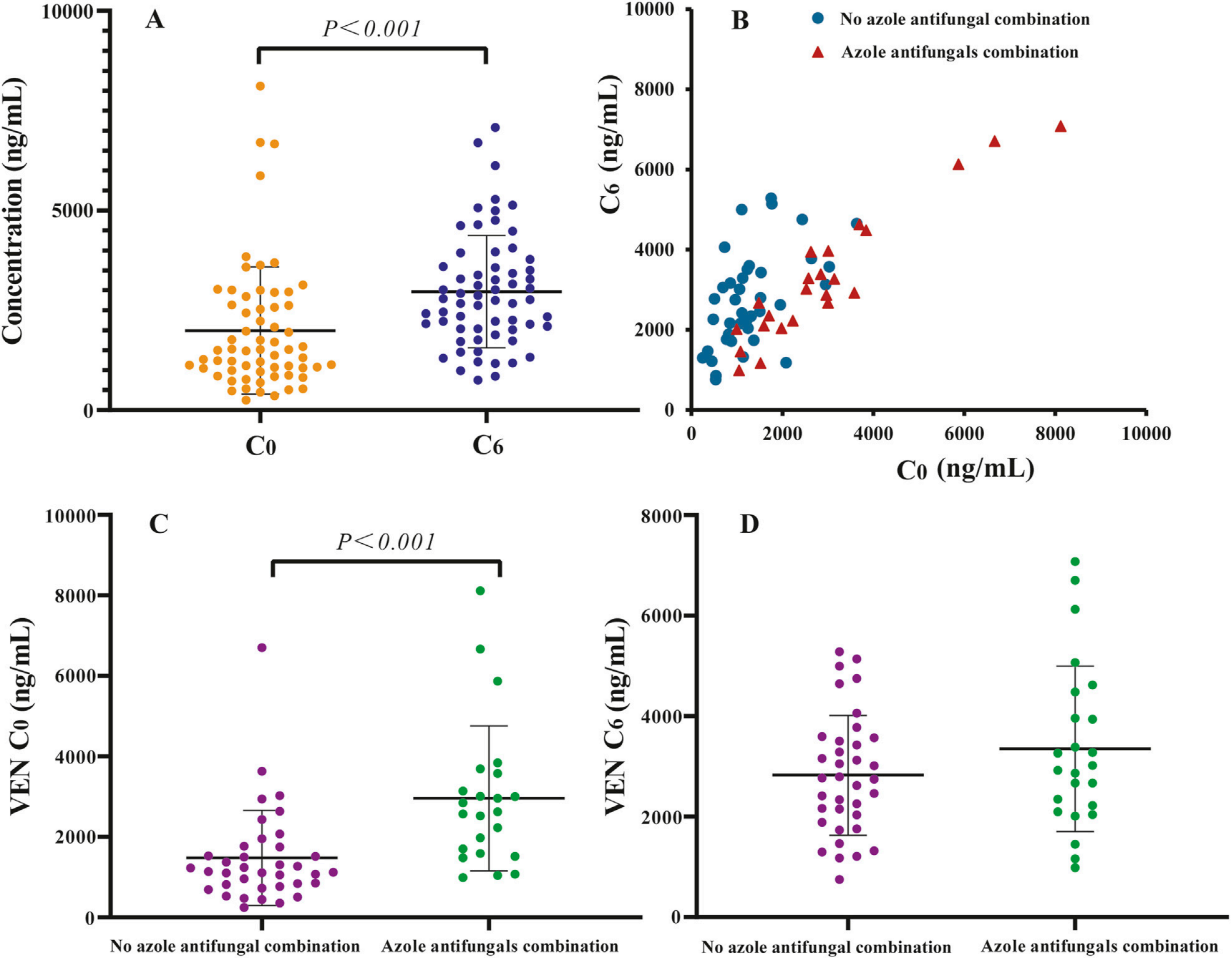
Distribution of venetoclax (VEN) trough steady-state plasma concentration (C_0_) and 6 h after oral dose plasma concentration (C_6_) in Chinese myelodysplastic syndromes (MDS) patients: **(A)** scatter plots of plasma concentration of VEN, **(B)** paired of C_0_ and C_6_, **(C)** C_0_ of VEN 400 mg without azole antifungals and VEN 100 mg with azole antifungals, **(D)** C_6_ of VEN 400 mg without azole antifungals and VEN 100 mg with azole antifungals.

The average C_0_ of VEN in Chinese MDS patients was 1990.60 ± 1,591.12 ng/mL, while the C_6_ was 2,966.66 ± 1,406.96 ng/mL (Figure 1A). Both C_0_ and C_6_ exhibited large interindividual variability, which is consistent with the results of previous studies (Yang et al., 2022; Kobayashi et al., 2022; De Gregori et al., 2023; Wang et al., 2024). Spearman’s test showed a moderate correlation between C_0_ and C_6_ (*r* = 0.605, Figure 1B). However, it is interesting that the C_6_ of eight patients was lower than their C_0_.

The potential influences associated with VEN concentration in Chinese MDS patients were analyzed. Univariate and multivariate analyses indicated that many factors, including age, gender, weight, albumin levels, liver and renal function, as well as types of HMAs, did not affect the C_0_ and C_6_ of VEN (*P* > 0.05), except for the coadministration of azole antifungals (see Supplementary Table S1).

Twenty-four patients received a VEN dose of 100 mg once daily combined with posaconazole or voriconazole. Forty patients used VEN without azole antifungals, of which 37 received a VEN dose of 400 mg, 1 patient received a dose of 200 mg and 2 patients received a dose of 300 mg. Mean VEN C_0_ and C_6_ were 1,479.29 ± 1,183.51 ng/mL and 2,826.05 ± 1,191.76 ng/mL for a 400 mg dose, 2,958.96 ± 1800.51 ng/mL and 3,349.00 ± 1,644.41 ng/mL for a 100 mg dose coadministered with azole antifungals, respectively (Figures 1C,D). Compared to 400 mg VEN without azole antifungals, coadministration of 100 mg VEN with posaconazole or voriconazole increased the mean VEN C_0_ and C_6_ by 100.03% and 18.50%, respectively. The average C_0_ of 100 mg VEN administered with azole antifungals was significantly higher than that C_0_ of 400 mg VEN administered without azole antifungals (*P* < 0.05).

### 3.3 Exposure‐efficacy analyses

Exposure-efficacy analyses were performed using data from patients underwent VEN + HMAs as the initial treatment or received 1 - 3 cycles of HMAs as prior regimens and added VEN within 4 cycles. Among the 49 candidates eligible for exposure-efficacy analyses, 10 did not undergo efficacy assessment. Consequently, data from 39 patients were available for analysis, with 5 cases (12.82%) at intermediate risk, 14 cases (35.90%) at high risk, and 20 cases (51.28%) at very-high risk. The findings demonstrate that within this patient cohort, the combination of VEN and HMAs has achieved an ORR of 69.23%. Subgroup analysis revealed that among the 33 naïve MDS patients, the ORR was 66.67% (22/33), with a CR rate of 15.15% (5/33) and an mCR rate of 45.45% (15/33). In 6 patients who received 1-3 cycles of HMAs as prior regimens, the ORR and CR of VEN therapy were 83.33% (5/6) and 33.33% (2/6), respectively.

**FIGURE 2:**
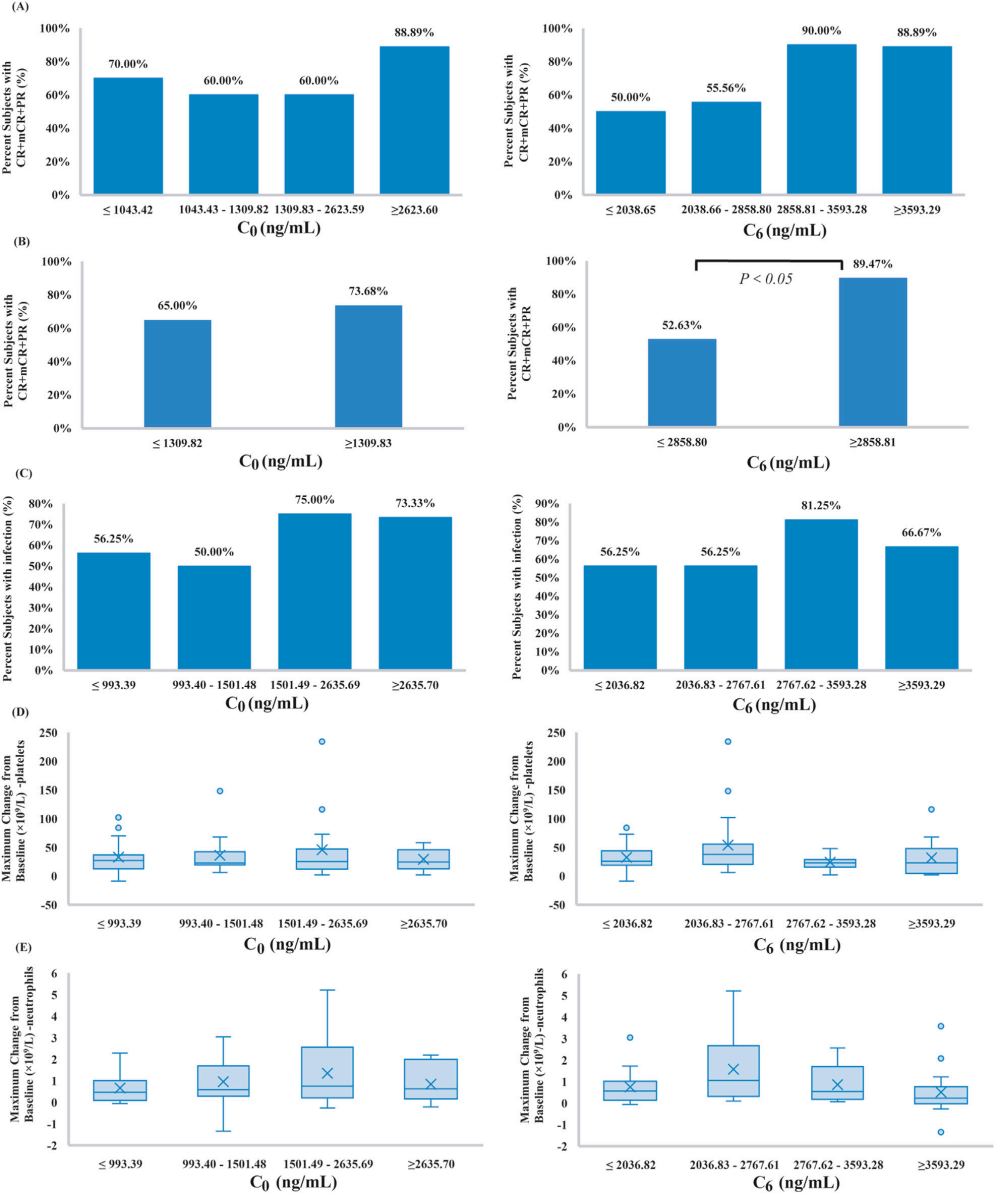
**(A,B)** Observed response rates for VEN plus hypomethylating agents (HMAs) by C_0_ and C_6_ quartiles and binary, **(C)** occurrence of observed treatment‐emergent grade 3 or worse infections for VEN plus HMAs by C_0_ and C_6_ quartiles, **(D,E)** the maximal change from baseline in platelet and neutrophil counts for VEN plus HMAs by C_0_ and C_6_ quartiles. Boxplots show the 25th and 75th percentile (upper and lower boundaries of box), median (solid lines in box), mean (x symbols), and distribution of individual cases (circles).

Quartile-based plots of VEN C_6_ suggested a trend toward higher response rates (CR + mCR + PR) with increasing VEN exposure, as shown in Figure 2A. However, no statistically significant differences were observed among the quartile groups (*P* > 0.05). In contrast, when patients were grouped using a binary VEN C_6_ cutoff, a statistically significant association was found between higher VEN exposure and the probability of response (52.63% vs. 89.47%, *P* < 0.05, Figure 2B). Graphical analysis of VEN C_0_ indicated that neither quartile-based nor binary-based plots revealed a significant exposure-efficacy relationship (*P* > 0.05).

**FIGURE 3:**
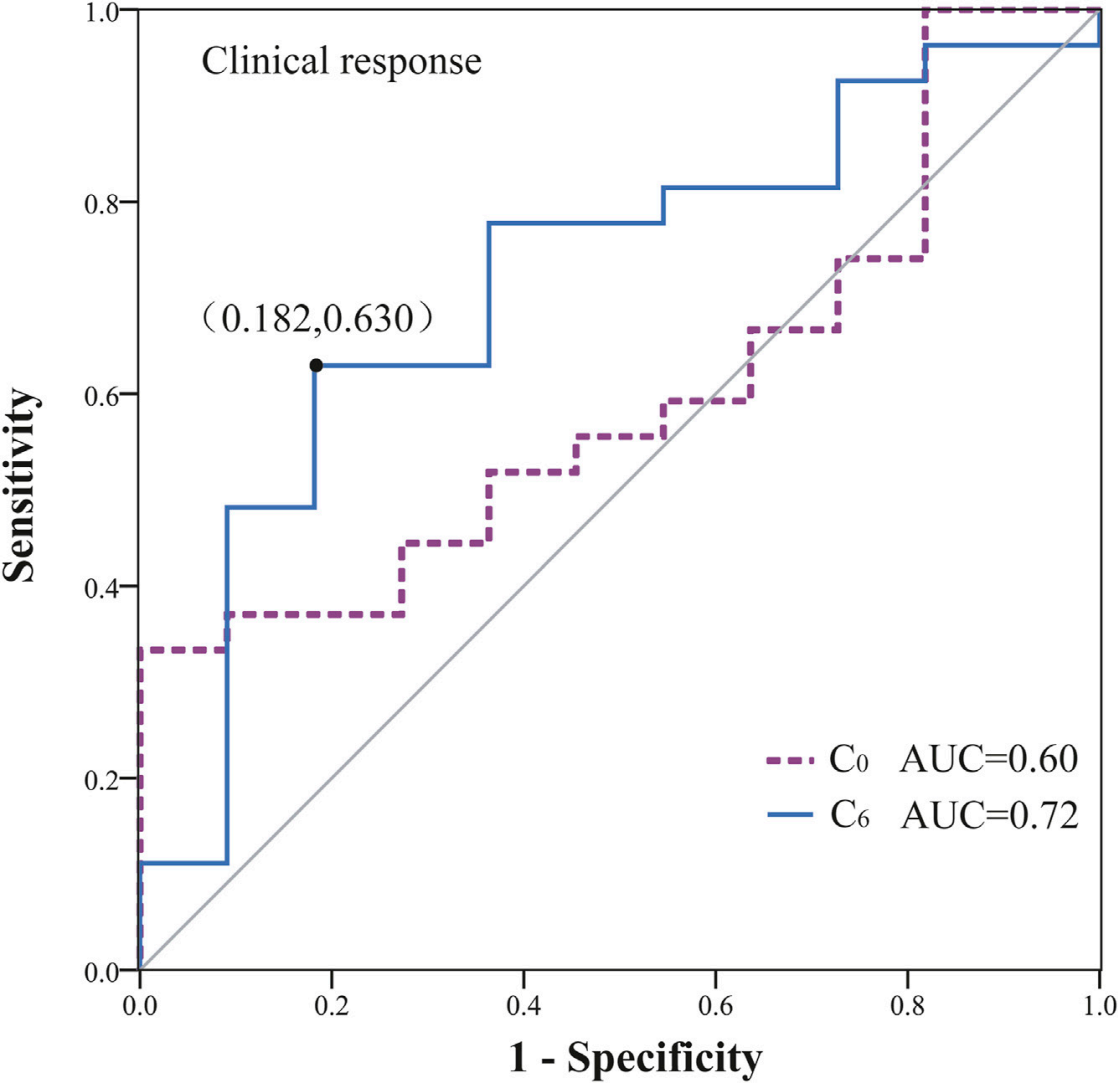
Receiver operator characteristic (ROC) curve for predicting treatment efficacy based on VEN concentrations (AUC: area under the ROC curve).

To further evaluate whether the plasma concentrations of VEN could predict efficacy, ROC curves were created. As shown in Figure 3, C_0_ (AUC: 0.60, 95% confidence interval [CI] 0.41–0.79) did not show an association with treatment success (*P* > 0.05). Even after removing the C_0_ values that were greater than C_6_, no correlation between C_0_ and treatment effectiveness was observed (AUC: 0.52, 95% CI 0.32 to 0.72, *P* > 0.05). By contrast, ROC curve showed that a level ≥2,858.80 ng/mL for C_6_ provided the optimal cut-off point, with a sensitivity of 62.96% (95% CI: 44.44%–81.48%) and a specificity of 81.82% (95% CI: 54.55% to >99.99%). Although the magnitude of the AUC was moderate (0.72; 95% CI 0.54–0.90), it was significantly greater than the null value of 0.5 (*P* < 0.05).

**FIGURE 4:**
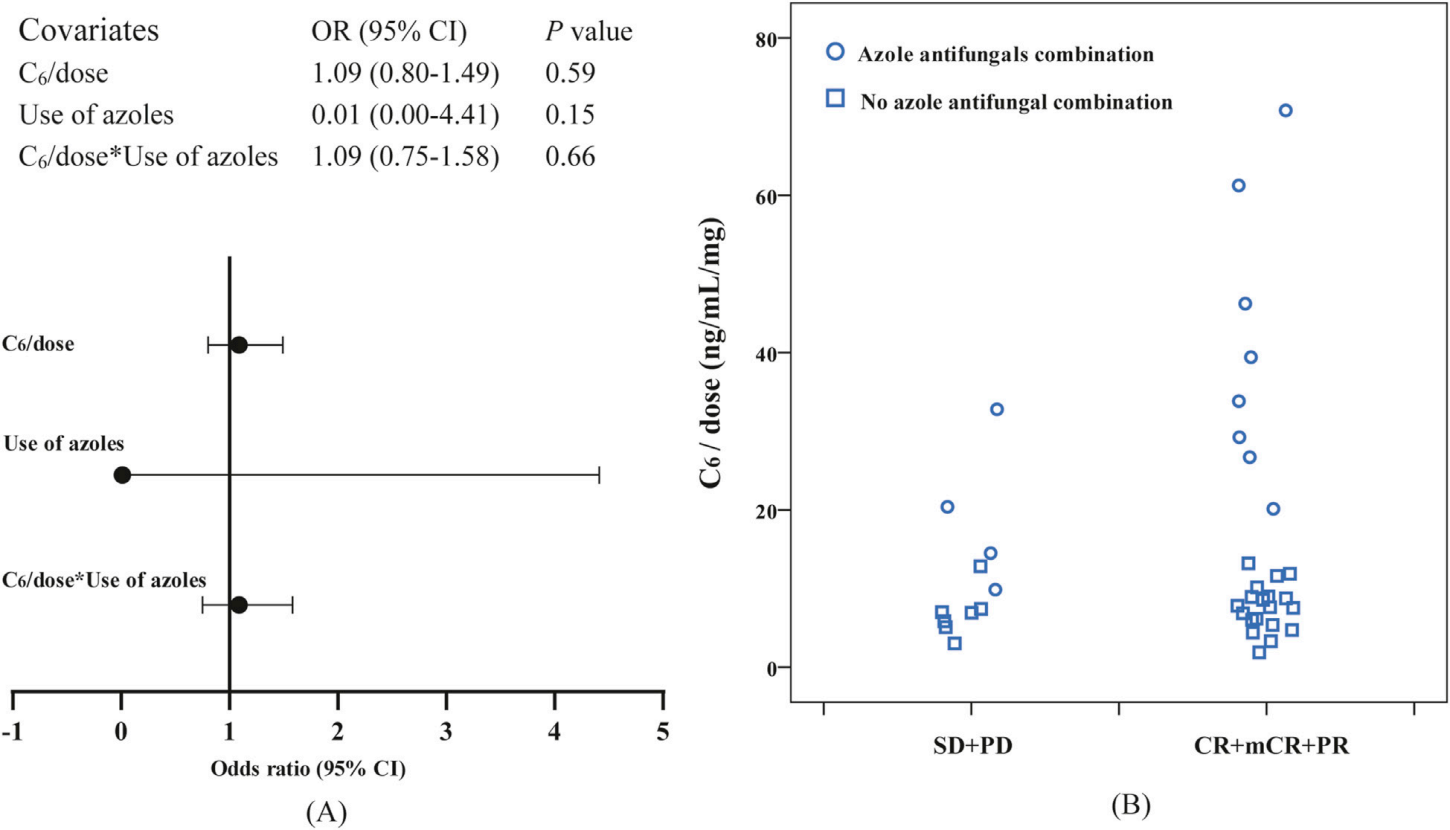
Observation of the correlation between dose-normalized C_6_ (C_6_/dose) and efficacy: **(A)** the interaction effect between C_6_/dose and azole coadministration in logistic regression, **(B)** a jittered scatter plot of C_6_/dose stratified by efficacy outcomes.

Logistic regression analysis demonstrated a significant association of C_6_ ≥ 2,858.80 ng/mL with the probability of marrow remission (*P* = 0.02, B: 2.035, odds ratio [OR]: 7.65, 95% CI: 1.37–42.71). Meanwhile, the bootstrap analysis confirmed the robustness of the parameter estimates for C_6_ ≥ 2,858.80 ng/mL in the final logistic regression model (*P* = 0.017, B: 2.035, OR: 7.65, BCa 95% CI: 0.03–23.13). Notably, after normalizing C_6_ for daily dose (C_6_/dose), the association with marrow remission was no longer statistically significant (*P* = 0.31, B: 0.03, OR: 1.03, 95% CI: 0.97–1.10), suggesting collinearity between low dose and azole coadministration in our cohort. Subsequent analyses were conducted to examine the interaction effect between C_6_/dose and azole coadministration, with the results depicted in Figure 4A; given the limited sample size, non-significant results were obtained. Figure 4B presents a jittered scatter plot of C_6_/dose stratified by efficacy outcomes, revealing that coadministration of azoles is associated with elevated C_6_/dose values and suggesting that the marrow remission group may exhibit higher C_6_/dose levels. Overall, current data are insufficient to determine whether per-mg exposure exerts an independent predictive effect once dose and inhibitor status are disentangled. No other covariates were significantly associated with efficacy outcomes (see Supplementary Table S2).

### 3.4 Exposure‐safety analyses

A total of 64 patients were included in the exposure-safety analyses, including 39 patients in the exposure-efficacy analyses. All patients experienced varying degrees of myelosuppression during the treatment cycle, with grade ≥3 neutropenia in 90.63% and grade ≥3 thrombocytopenia in 82.81% of patients. The most frequent non-hematological AE was grade ≥3 infection, which occurred in 41 (64.06%) patients.

Other non-hematological toxicities were relatively mild (grade 1–2) and infrequent (total: 45.31%). Gastrointestinal AEs occurred in 19 (29.69%) patients, mainly manifesting as nausea, vomiting, anorexia, abdominal pain, diarrhea, and constipation. In addition, four patients experienced pruritus (6.25%), while 15.63% of patients (10/64) had liver function test abnormalities probably related to VEN treatment. No tumorlysis syndrome or nephrotoxicity was observed in our cohort.

VEN exposure (C_0_ and C_6_) quartiles were used to observe the trend of the proportion of patients with grade ≥3 infection across the whole concentration range (Figure 2C). We noticed that higher VEN exposure tended to be associated with an increased risk of infection, although these were nonsignificant (*P* > 0.05). In ROC analyses, both C_0_ (complete dataset: AUC 0.62, 95% CI 0.48 to 0.77, *P* > 0.05; excluding C_0_ > C_6_ data: AUC 0.57, 95% CI 0.41 to 0.73, *P* > 0.05) and C_6_ (AUC 0.56, 95% CI 0.41 to 0.72, *P* > 0.05) of VEN were poor predictors of clinical infection; therefore, the optimal cut-off values of C_0_ and C_6_ could not be determined.

Among the hematological AE variables, the maximal change from baseline in platelet and neutrophil counts was evaluated (Figures 2D,E). According to the quartile plots, the rates of hematological variation tend to plateau in the C_0_ or C_6_ range studied. Kruskal-Wallis analysis showed that there was no evident relationship between VEN exposure and hematological AE variables (*P* > 0.05).

## 4 Discussion

Currently, approved therapies for MDS are limited in both number and efficacy, highlighting the need for novel strategies to improve patient outcomes. Preclinical studies showed that BCL-2 is overexpressed in high-risk MDS, and its inhibition induces apoptosis in MDS progenitor cells (Jilg et al., 2016; Parker et al., 2000). Since the FDA approved VEN for the treatment of AML, which shares many biological features with higher-risk MDS, off-label use of combination therapy with HMAs and VEN in MDS has become increasingly common. (Ball et al., 2020; Bazinet et al., 2022; Zeidan et al., 2023; Du et al., 2023; Azizi et al., 2020). This study investigated the *in vivo* exposure metrics of VEN in combination with HMAs for the treatment of MDS in Chinese patients to determine whether there is a correlation between VEN exposure and therapeutic efficacy or AEs. Such an understanding is crucial to further reflect on the potential requirement for TDM as a component of the routine clinical care of patients receiving VEN.

Notably, the relationships between VEN exposure and ORR or key safety end points were less definitive in previous studies involving other diseases. Some studies have failed to show the associations (Agarwal et al., 2019; Brackman et al., 2022; Samineni et al., 2022), on the contrary, in clinical studies involving patients with non-Hodgkin lymphomas, AML and chronic lymphocytic leukemia (CLL), a relationship between VEN exposure and a higher probability of response was observed (Parikh et al., 2018; Freise et al., 2017; Hagihara et al., 2024). The varying results are likely due to heterogeneity in the studied populations, inclusion of multiple VEN combination regimens, and different dosages of VEN administration.

In the current study, concentration-effect ROC curve analysis showed the discrimination potential of VEN C_6_ for effectiveness. A plasma threshold of 2,858.80 ng/mL was significantly associated with treatment success in Chinese MDS patients (*P* < 0.05). Our results indicated that the plasma exposure of VEN may be a key factor influencing therapeutic efficacy. In contrast, higher VEN C_0_ were not associated with an increase in effectiveness based on ROC curve analysis. Other important factors, including patient demographics and general disease characteristics such as the presence of a TP53, DNMT3A, RUNX1, ASXL1, DDX41 mutation, grade III/IV neutropenia at baseline, or number of prior therapies were not found to be related to the probability of marrow remission in the logistic regression analyses.

In the exposure-safety analyses, we were unable to demonstrate any statistically significant relationships between C_0_ and C_6_ of VEN and various safety outcomes (i.e., grade ≥3 infection or myelosuppression). Based on graphical analyses, increases in VEN C_0_ seem to be associated with a trend of an increase in the infection rate, but it was not statistically significant (*P* > 0.05). On the other hand, the maximal change from baseline in platelet and neutrophil counts appeared to be near maximal across the VEN concentration range corresponding to the therapeutic doses. The results of this study indicated that both hematological AEs and non-hematological AEs of VEN in Chinese MDS patients do not show the feature of concentration dependence.

Besides the results of exposure-response analysis, some other findings also hold clinical interest and should be taken into consideration. First, hematological AEs were the most common toxicities observed, consistent with prior reports (Zeidan et al., 2023; Wei et al., 2019; DiNardo et al., 2019; Maiti et al., 2019). Since our study population consisted of MDS patients, certain hematological AEs occurred prior to VEN initiation and were attributed to underlying disease. Therefore, our patients showed a higher rate of hematological AEs than those in some previous reports (Zeidan et al., 2023; Wei et al., 2019; DiNardo et al., 2019; Maiti et al., 2019). Interestingly, Kobayashi *et al.* reported that common grade 3/4 AEs in Japanese patients included decreased white blood cell count (91.7%), thrombocytopenia (83.3%), and febrile neutropenia (66.6%), which is consistent with our data (Kobayashi et al., 2022). Further, Yamamoto *et al.* reported that Japanese patients exhibited a higher incidence of hematological AEs in the VIALE-A trial subgroup analysis (Yamamoto et al., 2022), suggesting that racial differences (Asian vs. non‐Asian) might influence AE occurrence.

VEN is a P-glycoprotein (P-gp) substrate and is eliminated predominantly via CYP3A4 enzymes. Therefore, the second issue is the influence of drug-drug interactions. Many patients with MDS undergoing VEN-based therapy are immunocompromised, and azole antifungals are often used. However, azole compounds carry the risk of drug-drug interactions because of their effects on CYP3A4 and transporter proteins (e.g., P-gp) (Hagihara et al., 2024; Kawedia et al., 2025). In CLL patients, the current US prescribing information recommends at least a 75% reduction in VEN dose (≤100 mg) when coadministered with strong CYP3A inhibitors, such as posaconazole. (AbbVie Inc. & Genentech USA, 2016). In the present study, concomitant use of azole antifungals with VEN 100 mg resulted in 100.03% and 18.50% higher VEN C_0_ and C_6_, compared with VEN 400 mg administered without azole antifungals. The increase in C_6_ observed in this study was considerably lower than the 86% indicated in the prescribing information and the 93% reported in the literature. (AbbVie Inc. & Genentech USA, 2016; Agarwal et al., 2017). However, Gao et al. observed that azole antifungals were able to increase the peak concentration of VEN 100 mg to the same level as VEN 400 mg administered without azole antifungals, but the C_0_ of VEN 100 mg was much higher than that of the 400 mg (Gao et al., 2023), which is highly consistent with our results. Additionally, the research conducted by Wen *et al.* similarly noted this phenomenon (Wen et al., 2024). Both our study and two others focused on Chinese patients; thus, racial differences may account for the C_6_ disparity.

Based on the phase Ib clinical trial of VEN-HMAs (NCT02203773) and critical pharmacokinetic studies, the most common range of trough and peak concentrations for VEN was 500–4,000 ng/mL and 2,000–5,500 ng/mL, respectively, which is consistent with the present results. (Yang et al., 2022; Wang et al., 2024; Brackman et al., 2022; Agarwal et al., 2017; DiNardo et al., 2018). However, it is noteworthy that eight paired C_0_-C_6_ measurements showed lower C_6_ levels than C_0_ in our study. We speculate that the C_0_-C_6_ inversion may be caused by fluctuations in the timing of the C_0_ sample collection, as well as by the influence of azole antifungals on the concentration-time curve of VEN. The TDM protocol specifies that blood samples should be collected within a ±10 min window of the designated time point. Nevertheless, the actual C_0_ sampling time may be earlier than anticipated due to several factors, including the batch collection of various laboratory samples and fluctuations in patients' meal schedules. Compared to C_0_, the sampling timing for C_6_ is more strictly enforced in real-world clinical practice.

Ultimately, the majority of patients in this study were very high or high-risk (87.50%, IPSS-R > 4.5) MDS, and 92.19% were MDS-EB subtypes, suggesting a poor prognosis. Nevertheless, the combination of VEN and HMAs achieved a high rate of marrow remission, demonstrating effectiveness in both treatment-naïve and treatment-experienced MDS populations. The high remission rate achieved through the combination of VEN and HMAs treatment regimen provides more patients with the opportunity to undergo HSCT. In this study, 18.75% of the patients received HSCT at our institution.

The present study has some limitations that should be highlighted. First, our study was retrospective and limited by the small numbers of patients, heterogeneous patient population, and short follow-up period; a larger sample size would enhance the robustness of our findings. Second, uncertainties are inherent in real-world retrospective studies, particularly concerning the precision of blood sampling times and the effects of food on drug absorption rates, which could lead to variations in drug exposure. Finally, the exposure-response relationship observed for C_0_ and C_6_ in this study does not fully capture the dynamics across the entire concentration-time curve (AUC_0-τ_). In sparse sampling, establishing a population pharmacokinetic model to predict the AUC of VEN for exposure-response analysis is an excellent choice. It is essential to pursue more extensive research in this domain to advance our understanding further.

## 5 Conclusion

The average C_0_ of VEN in Chinese MDS patients was 1990.60 ± 1,591.12 ng/mL, while the C_6_ was 2,966.66 ± 1,406.96 ng/mL. Both C_0_ and C_6_ exhibited significant interindividual variabilities. The concurrent use of azole antifungals was identified as the only factor that influenced VEN concentrations. Notably, even with a reduced dosage of VEN, the average C_0_ of 100 mg VEN coadministered with azole antifungals was significantly higher than that of 400 mg VEN administered without azole antifungals. Based on logistic regression and ROC curve analyses, the peak plasma concentration of VEN, without dose normalization, exhibits a significant correlation with treatment success (*P* < 0.05). Other factors, including C_0_, patient demographics, and general disease characteristics such as the presence of one or more molecular mutations, grade III/IV neutropenia at baseline, or number of prior therapies, were not found to be related to the probability of marrow remission. In safety analyses, higher VEN concentrations were not associated with an increased probability of grade ≥3 infection or a more serious decrease in platelets and neutrophils, indicating that these evaluated safety endpoints were not concentration-limiting. Our retrospective real-world data suggest that TDM of VEN may improve treatment outcomes in Chinese MDS patients. Future research is necessary to conduct more in-depth and large-scale analyses to determine the optimal exposure threshold for VEN.

## Data Availability

The original contributions presented in the study are included in the article/Supplementary Material, further inquiries can be directed to the corresponding authors.
