# Supplementary material for: Exposure-response analyses of venetoclax combined with hypomethylating agents in myelodysplastic syndromes: a retrospective study
Source: Front Pharmacol. 2025 Jul 14;16:1586910. doi: 10.3389/fphar.2025.1586910 (PMC12301662; doi:10.3389/fphar.2025.1586910)
Supplement: Supplementary file 1 [file Supplementaryfile1.docx]

Supplementary Material

# Supplementary Figures and Tables

##
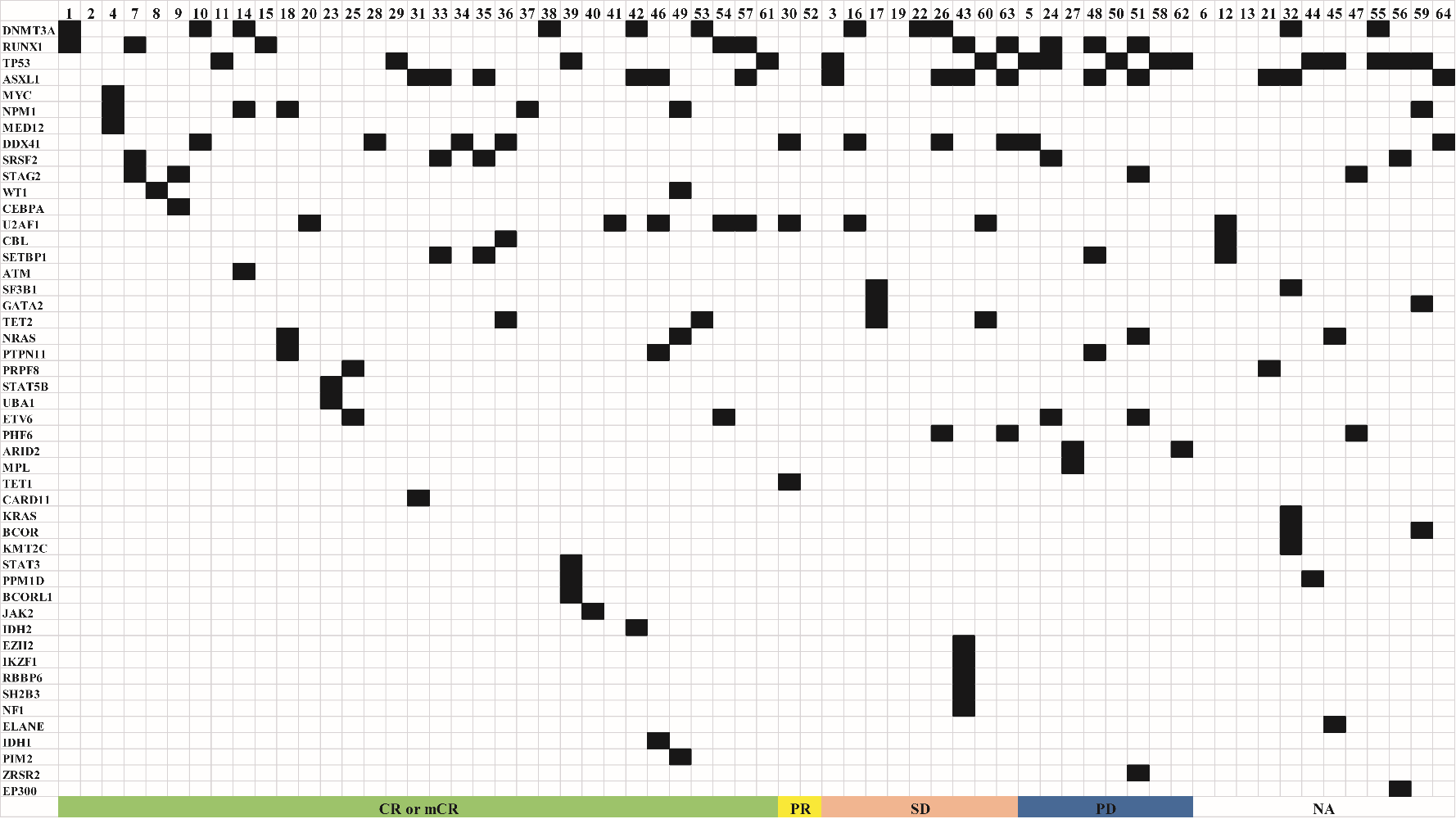
Supplementary Figures

## Supplementary Figure 1. Molecular mutation in 64 patients and the efficacy of VEN + HMAs treatment (CR: complete remission, PR: partial response, mCR: marrow CR, SD: stable disease, PD: progressive disease, NA: not available).

## Supplementary Tables

**Supplementary Table 1.** Univariate and multivariate analyses of factors affecting VEN C_0_ and C_6_.

| Factors | C_0_ | | |  | | C_6_ | |
| --- | --- | --- | --- | --- | --- | --- | --- |
|  | B (95% CI) | *P* value |  | | B (95% CI) | | *P* value |
| **Univariate analysis** | | | | | | | |
| Use of azole antifungals | 1479.672 (709.609 - 2249.735) | **0.000** |  | | 522.956 (-209.751 - 1255.663) | | 0.158 |
| Male vs. female | -185.134 (-1034.527 - 664.260) | 0.664 |  | | -314.591 (-1055.479 - 426.298) | | 0.722 |
| Age | 16.962 (-20.367 - 54.291) | 0.367 |  | | 23.537 (-8.662 - 55.736) | | 0.149 |
| BMI | -54.602 (-141.610 - 32.405) | 0.214 |  | | -63.323 (-138.371 - 11.724) | | 0.097 |
| ALB | -80.913 (-199.197 - 37.372) | 0.176 |  | | -2.584 (-106.795 - 101.627) | | 0.961 |
| Cr | -3.195 (-22.735 - 16.345) | 0.745 |  | | 1.312 (-15.813 - 18.438) | | 0.879 |
| AST | 27.980 (-13.975 - 69.936) | 0.187 |  | | 7.287 (-29.458 - 44.031) | | 0.693 |
| Kind of HMAs | -546.634 (-1585.353 - 492.085) | 0.297 |  | | -322.329 (-1231.423 - 586.765) | | 0.481 |
| **Multivariate analysis** | | | | | | | |
| Use of azole antifungals | 1334.087 (431.622 – 2236.551) | **0.005** |  | | 572.283 (-278.029 – 1422.595) | | 0.183 |
| Male vs. female | -452.054 (-1411.276 - 507.168) | 0.349 |  | | -647.660 (-1548.934 - 253.614) | | 0.155 |
| Age | 21.248 (-14.879 - 57.376) | 0.243 |  | | 23.133 (-10.936 - 57.201) | | 0.179 |
| BMI | -29.998 (-114.537 - 54.540) | 0.479 |  | | -51.996 (-131.660 - 27.668) | | 0.196 |
| ALB | -22.427 (-142.816 - 97.961) | 0.710 |  | | 24.950 (-87.925 - 137.825) | | 0.659 |
| Cr | 1.619 (-21.117 - 24.355) | 0.887 |  | | 8.131 (-13.254 - 29.517) | | 0.449 |
| AST | 23.852 (-18.799 - 66.502) | 0.267 |  | | 7.291 (-32.570 - 47.151) | | 0.715 |
| Kind of HMAs | -193.231 (-1218.961 - 832.500) | 0.707 |  | | -60.673 (-1030.078 - 908.732) | | 0.901 |

C_0_, pre-dose concentration; C_6_, 6 hours after oral dose plasma concentration; ALB, albumin; Cr, creatinine value; AST, aspartate transaminase; BMI, body mass index; VEN, venetoclax; HMAs, hypomethylating agents; B, unstandardized coefficient; CI, confidence interval.

**Supplementary Table 2.** Univariate and multivariate analyses of the factors associated with the efficacy of VEN.

| Factors | OR | 95% CI | *P* value |
| --- | --- | --- | --- |
| **Univariate analysis** |  |  |  |
| Age |  |  |  |
| <60 years | 1 |  |  |
| ≥60 years | 0.963 | 0.242 - 3.829 | 0.957 |
| Body mass index |  |  |  |
| <25 | 1 |  |  |
| ≥25 | 1.750 | 0.442 - 6.928 | 0.425 |
| Gender |  |  |  |
| Female | 1 |  |  |
| Male | 1.077 | 0.276 - 4.197 | 0.915 |
| Grade III/IV neutropenia at baseline |  |  |  |
| No | 1 |  |  |
| Yes | 1.077 | 0.276 - 4.197 | 0.915 |
| DNMT3A mutation |  |  |  |
| No | 1 |  |  |
| Yes | 0.682 | 0.134 - 3.473 | 0.645 |
| RUNX1 mutation |  |  |  |
| No | 1 |  |  |
| Yes | 0.375 | 0.064 - 2.211 | 0.279 |
| TP53 mutation |  |  |  |
| No | 1 |  |  |
| Yes | 0.240 | 0.034 - 1.678 | **0.150** |
| ASXL1 mutation |  |  |  |
| No | 1 |  |  |
| Yes | 0.375 | 0.064 - 2.211 | 0.279 |
| DDX41 mutation |  |  |  |
| No | 1 |  |  |
| Yes | 0.870 | 0.136 - 5.545 | 0.882 |
| Three or more molecular mutations |  |  |  |
| No | 1 |  |  |
| Yes | 0.663 | 0.168 - 2.620 | 0.558 |
| C_6_ of VEN |  |  |  |
| <2858 ng/mL | 1 |  |  |
| ≥2858 ng/mL | 7.650 | 1.370 - 42.713 | **0.020** |
| Prior therapies |  |  |  |
| No | 1 |  |  |
| Yes | 2.500 | 0.259 - 24.096 | 0.428 |
| **Multivariate analysis** |  |  |  |
| C_6_ of VEN |  |  |  |
| <2858 ng/mL | 1 |  |  |
| ≥2858 ng/mL | 8.094 | 1.334 - 49.114 | **0.023** |
| TP53 mutation |  |  |  |
| No | 1 |  |  |
| Yes | 0.189 | 0.020 - 1.781 | 0.146 |
